# Novel homozygous variants in *PRORP* expand the genotypic spectrum of combined oxidative phosphorylation deficiency 54

**DOI:** 10.1038/s41431-023-01437-2

**Published:** 2023-08-09

**Authors:** Thomas B. Smith, Alessandro Rea, Huw B. Thomas, Kyle Thompson, Monika Oláhová, Reza Maroofian, Mina Zamani, Langping He, Saeid Sadeghian, Hamid Galehdari, Nava Shaul Lotan, Tal Gilboa, Kristin C. Herman, Thomas J. McCorvie, Wyatt W. Yue, Henry Houlden, Robert W. Taylor, William G. Newman, Raymond T. O’Keefe

**Affiliations:** 1 Division of Evolution, Infection and Genomics, School of Biological Sciences, University of Manchester, Manchester, M13 9PL UK; 2 Manchester Centre for Genomic Medicine, St Mary’s Hospital, Manchester University NHS Foundation Trust, Manchester, M13 9WL UK; 3 Wellcome Centre for Mitochondrial Research, Clinical and Translational Research Institute, Faculty of Medical Sciences, Newcastle University, Newcastle upon Tyne, NE2 4HH UK; 4 Department of Applied Sciences, Faculty of Health & Life Sciences, Northumbria University, Newcastle upon Tyne, UK; 5 Department of Molecular Neuroscience, UCL Queen Square Institute of Neurology, London, WC1N 3BG UK; 6 Department of Biology, Faculty of Science, Shahid Chamran University of Ahvaz, Ahvaz, Iran; 7 Narges Medical Genetics and Prenatal Diagnosis Laboratory, Kianpars, Ahvaz, Iran; 8 NHS Highly Specialised Service for Rare Mitochondrial Disorders, Newcastle upon Tyne Hospitals NHS Foundation Trust, Newcastle upon Tyne, NE1 4LP UK; 9 Department of Pediatric Neurology, Golestan Medical, Educational, and Research Center, Ahvaz Jundishapur University of Medical Sciences, Ahvaz, Iran; 10 Genetic Department, Hadassah Hebrew University Hospital, Jerusalem, Israel; 11 Pediatric Neurology, Hadassah Hebrew University Hospital, Jerusalem, Israel; 12 UC Davis Medical Center MIND Institute, 2825 50th Street, Sacramento, CA 95817 USA; 13 Biosciences Institute, Faculty of Medical Sciences, Newcastle University, Newcastle upon Tyne, NE2 4HH UK

**Keywords:** Medical genomics, Genetic testing, Infertility, Translational research

## Abstract

Biallelic hypomorphic variants in *PRORP* have been recently described as causing the autosomal recessive disorder combined oxidative phosphorylation deficiency type 54 (COXPD54). COXPD54 encompasses a phenotypic spectrum of sensorineural hearing loss and ovarian insufficiency (Perrault syndrome) to leukodystrophy. Here, we report three additional families with homozygous missense *PRORP* variants with pleiotropic phenotypes. Each missense variant altered a highly conserved residue within the metallonuclease domain. In vitro mitochondrial tRNA processing assays with recombinant TRMT10C, SDR5C1 and PRORP indicated two COXPD54-associated *PRORP* variants, c.1159A>G (p.Thr387Ala) and c.1241C>T (p.Ala414Val), decreased pre-tRNA^Ile^ cleavage, consistent with both variants impacting tRNA processing. No significant decrease in tRNA processing was observed with *PRORP* c.1093T>C (p.Tyr365His), which was identified in an individual with leukodystrophy. These data provide independent evidence that *PRORP* variants are associated with COXPD54 and that the assessment of 5′ leader mitochondrial tRNA processing is a valuable assay for the functional analysis and clinical interpretation of novel *PRORP* variants.

## Introduction

Biallelic variants in Protein Only RNase P Catalytic Subunit (*PRORP)* have recently been associated with a newly defined syndrome, combined oxidative phosphorylation deficiency 54 (COXPD54) (MIM #619737), encompassing a phenotypic spectrum of Perrault syndrome and leukodystrophy [1]. Perrault syndrome (MIM #233400) is a rare, autosomal recessive, clinically and genetically heterogeneous disorder characterised by bilateral sensorineural hearing loss (SNHL) in both sexes and primary ovarian insufficiency in 46, XX karyotype females [2, 3]. The individuals with biallelic *PRORP* variants, reported to date, presented with pleiotropic phenotypes ranging in clinical severity, with PRORP variant protein resulting in reduced mitochondrial tRNA processing in vitro [1]. With the small number of reported affected individuals with COXPD54, genotype–phenotype correlations have yet to be established and independently replicated. PRORP (MRPP3) is one of the three subunits constituting the human mitochondrial RNase P (mtRNase P) complex with MRPP1 (TRMT10C) and MRPP2/SDR5C1 (HSD17B10). This multimeric complex aids mitochondrial tRNA maturation by catalysing endonucleolytic cleavage of the 5′ leader sequence from polycistronic mitochondrial RNA transcripts [4].

Here, we present three further unrelated families with homozygous, missense *PRORP* variants. Consistent with the previously reported cases, the variants are within the functional metallonuclease domain and alter highly conserved residues [1]. The affected individuals have phenotypes overlapping with the previous reports and provide evidence that the clinical severity correlates with the degree of mitochondrial tRNA processing deficit. These findings provide independent supportive evidence for the association of biallelic *PRORP* variants with defective mitochondrial tRNA processing, resulting in a phenotypic spectrum encompassing Perrault syndrome and COXPD54.

## Materials and methods

### Variant identification methodology

Biallelic variants in *PRORP* were identified by exome sequence analysis in all three families (see Supplementary information). Segregation analysis was undertaken using Sanger sequencing for all unaffected and affected relatives where available. Details of respiratory chain activity assays, immunoblotting and mitochondrial tRNA processing assays are also outlined in detail in the Supplementary information.

## Results

The three probands presented with diverse clinical phenotypes outlined below. Additional clinical, in vitro and protein modelling data is available in the Supplementary information.

**Fig. 1:**
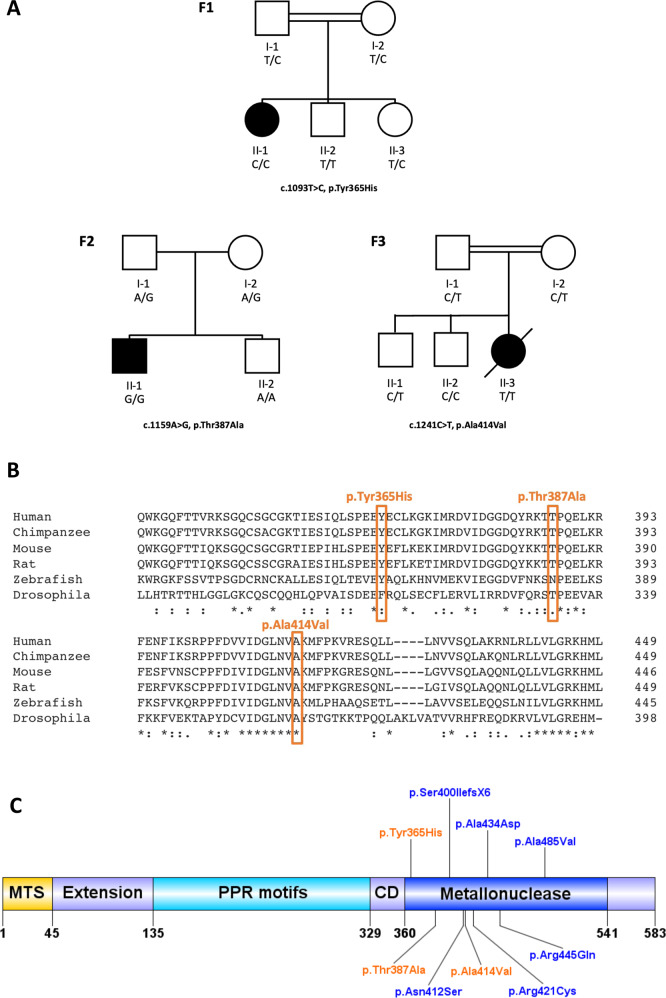
*PRORP* family pedigrees, conservation of *PRORP* variant residues across a selection of species and *PRORP* variant locations. **A** Pedigrees for families F1–F3, each with homozygous *PRORP* variants in the proband. **B** Position of variant residues highlighted in orange. Symbols below the sequence alignments represent level of conservation across species, with * (asterisk) indicating full conservation, : (colon) indicating strongly similar properties, . (period) indicating weakly similar properties and no symbol indicating no conservation. Visualised using Clustal Omega alignment software. **C** Schematic illustrating the location of all established *PRORP* variants to date. Novel variants are coloured in orange, whilst previously published variants are in blue. Created using DOG (Domain Graph) software [9]. MTS mitochondrial targeting sequence, PPR pentatricopeptide repeat, CD central domain.

Family F1 is a consanguineous family with a 28-year-old female proband from Iran who presented with mild intellectual impairment, gait abnormality, behavioural problems and hypothyroidism. There is no evidence of SNHL or ovarian insufficiency; however, brain MRI demonstrates cerebellar atrophy and multifocal leukoencephalopathy (Supplementary Fig. S1A). Menstruation is irregular (once every 4–6 months), and she has bilateral polycystic ovaries. Biochemical tests of ovarian function revealed hormone levels were within follicular phase reference values (Supplementary Fig. S1B). Exome sequencing revealed a homozygous missense *PRORP* (NM_014672.4:c.1093T>C (p.Tyr365His)) variant in the proband. The parents of the proband are heterozygous and her two clinically unaffected siblings, a brother and sister, are wildtype and heterozygous, respectively (Fig. 1A).

The proband from family F2 is a 7-year-old American male of Mexican background. He presented with severe to profound bilateral SNHL (Supplementary Fig. S1C), undergoing assessment aged 3 years, exhibiting global developmental delay, spastic diplegia and truncal hypotonia. He is also both nonverbal and non-ambulatory. Brain MRI displayed no white matter lesions. Trio exome sequencing of the proband revealed a homozygous variant in *PRORP* (NM_014672.4:c.1159A>G (p.Thr387Ala)), with no other candidate variants identified. Both parents are heterozygous for this variant and unaffected, whilst his healthy younger sibling is homozygous for the reference allele.

The proband from family F3 is the daughter of a consanguineous couple of Arab Muslim background from Israel. She was affected by isovaleric acidemia detected by newborn screening and homozygous for the *IVD*: c.941C>T;p.(Ala314Val) hypomorphic variant [5, 6]. The proband did not pass the neonatal hearing screening test on the right ear; however, a formal hearing test was not performed. She presented at 3.5 months with episodes of lactic academia (up to 11.5 mmol/l, normal range 2–4 mmol/l), preceded by a febrile infection, and was additionally diagnosed with atrial septal defect and severe pulmonary hypertension. She had repeatedly high lactate levels (2.6–6.4 mmol/l). She was affected by hypotonia, developmental delay and severe failure to thrive. Aged 1 year, she suffered a lactic acidosis crisis (8.4 mmol/l) with status epilepticus, after which she experienced epilepsy, extrapyramidal movement disorder and loss of developmental skills. She died at age 19 months. Brain MRI scans revealed cerebrospinal space dilation and diffuse restrictive changes in the perirolandic region and basal ganglia (Supplementary Fig. S1D). Exome sequencing identified the homozygous *PRORP* (NM_014672.4: c.1241C>T (p.Ala414Val)) variant. Both parents are heterozygous for the variant.

All three novel variants alter highly conserved residues (Fig. 1B) and are predicted deleterious by in silico analyses (Supplementary Table S2). The three variants are also absent from gnomAD, providing supportive evidence for pathogenicity [7].

**Fig. 2:**
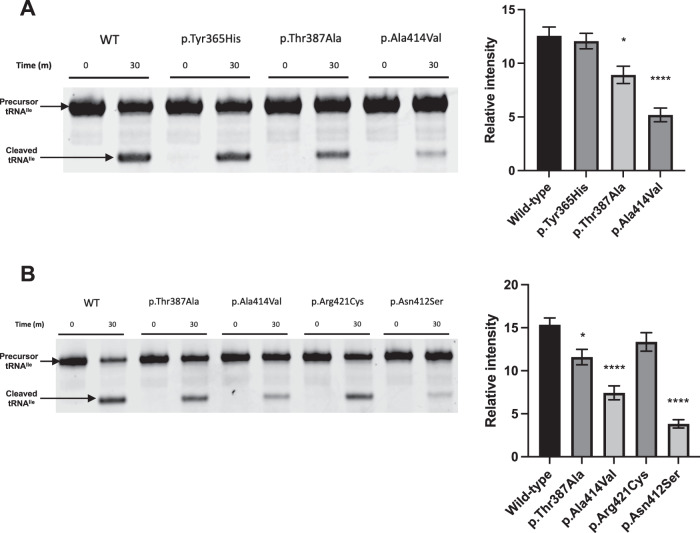
Functional assessment of *PRORP* variants in tRNA processing assays with mtRNase P. **A** Cleavage of the pre-tRNA^Ile^ 5′ leader sequence by mtRNase P containing wildtype (WT) or variant PRORP. The relative intensity of the pre-tRNA^Ile^ cleavage product was quantified with error bars representing the standard error of the mean. *N* = 4, *p < 0.05, ****p < 0.0001, one-way ANOVA with Dunnett’s multiple comparisons test, comparing wildtype to variants. **B** Comparison of mtRNase P activity to previously published *PRORP* variants. *N* = 5, *p < 0.05, ****p < 0.0001, one-way ANOVA with Dunnett’s multiple comparisons test, comparing wildtype to variants.

To assess whether the novel *PRORP* variants altered mitochondrial tRNA processing, we purified recombinant PRORP with the disease-associated variants and tested the endonucleolytic activity of the mtRNase P complex in the presence of pre-tRNA^Ile^ in vitro (Supplementary Fig. S3). After incubation with pre-tRNA^Ile^, mtRNase P complex with the p.Thr387Ala (F2) or p.Ala414Val (F3) PRORP variants significantly diminished the levels of 5′ cleavage product (*p* = 0.0121 and <0.0001 respectively) in comparison to wildtype PRORP (Fig. 2). The percentage decreases in relative intensity were 29% and 59%, respectively. The p.Tyr365His PRORP variant had little impact on 5′ leader processing, with a modest 4% decrease (*p* = 0.9330).

We also independently compared the novel p.Thr387Ala and p.Ala414Val variants to a disease-associated *PRORP* variant to assess the reproducibility of the tRNA processing assay [1]. The p.Arg421Cys and p.Asn412Ser *PRORP* variants were evaluated in the tRNA processing assay because they altered the cleavage of pre-tRNA^Ile^ to variable degrees [1]. The novel p.Thr387Ala and p.Ala414Val *PRORP* variants significantly reduced cleavage of the mtRNase P complex by 25% and 52% (*p* = 0.0153 and <0.0001), whilst the p.Arg421Cys and p.Asn412Ser variants diminished cleavage of the mtRNase P complex by 13% and 75% (*p* = 0.2873 and <0.0001) respectively, comparable to values in the previous report [1]. The variants p.Thr387Ala and p.Ala414Val were subsequently classified according to ACMG guidelines as likely pathogenic, whereas p.Tyr365His was classified as a variant of uncertain significance (VUS), as submitted to ClinVar.

## Discussion

Biallelic *PRORP* variants have been associated with diverse, overlapping pleiotropic phenotypes, with variable defects in mitochondrial tRNA processing [1]. The aim of this work was to independently confirm that novel *PRORP* variants identified in additional families are pathogenic and associated with the COXPD54 phenotypic spectrum.

Because all novel variants were located within the functional metallonuclease domain (Fig. 1C), we investigated the ability of mtRNase P complexes with *PRORP* variants to conduct 5′ leader processing of pre-tRNA^Ile^. mtRNase P complexes with the novel PRORP (p.Thr387Ala and p.Ala414Val) variants significantly decreased the intensity of the cleavage product compared to wildtype, indicating the function of the mtRNase P complex was impaired. The defect in 5′ leader processing was more pronounced with the variant from the F3 proband with a more severe phenotype. Fibroblasts from the affected individual in F3 demonstrated deficiencies in complex I activity (Supplementary Fig. S2B), highlighting a respiratory chain defect.

In contrast, mtRNase P complexes with PRORP p.Tyr365His did not significantly reduce pre-tRNA^Ile^ 5′ end cleavage. There are potential explanations for this finding. For example, protein modelling demonstrates that amino acid 365 is not located in the vicinity of the active site or any interaction regions (Supplementary Fig. S4). Furthermore, residues surrounding the amino acid are less conserved than the residues proximate to other disease-associated variants (Fig. 1). These observations indicate this region of the protein may be less essential for regular endonucleolytic function. The p.Tyr365His variant may also marginally reduce mtRNase P processing efficacy to levels which are difficult to detect experimentally but could still diminish processing in specific tissues. It is also possible that a different pathogenic mechanism is responsible for this phenotype, or that this variant does not cause COXPD54.

With the discovery of additional affected families, comparisons can begin to be made between *PRORP* variants and associated phenotypes (Supplementary Table S3). The proband with the p.Tyr365His PRORP VUS has a similar presentation to the individuals in the initial discovery study with the p.Arg421Cys PRORP variant [1]. Affected individuals in both families presented with leukodystrophy, mild learning disabilities and behavioural abnormalities, with no hearing impairment or ovarian insufficiency. Both variants also had little impact on mitochondrial tRNA processing. The probands from F3 in this study (PRORP p.Ala414Val) and P3 in the discovery study (PRORP p.Arg445Gln;p.Ser400Ilefs*6) were also comparable, presenting with severe childhood-onset features, including lactic acidosis, hypotonia, and seizures with white matter changes. Both missense variants reduced the levels of cleaved pre-tRNA^Ile^ in vitro, suggesting a relationship of increased phenotypic severity correlated with diminished mtRNase P function.

The p.Ala414Val PRORP variant reported here is in close proximity to the variant p.Asn412Ser reported previously [1], with both variants resulting in a significant decrease in cleavage product. Interestingly, the phenotype in individual P2 in the previous report [1] of isolated SNHL was less severe than that observed in F3 in this study. This difference in phenotypic severity could be because the F3 proband is homozygous for the p.Ala414Val variant, whereas individual P2 was compound heterozygous for p.Asn412Ser and the less deleterious c.1301C>A (p.Ala434Asp) *PRORP* variant.

Utilising the ClinGen scoring criteria for gene-disease validity [8], the initial *PRORP* discovery paper scored 9.75, which is concordant with moderate evidence for disease association. With the addition of our families, the score was increased to 12, upgrading the gene-disease association to strong. A third independent report is required for definitive confirmation that *PRORP* variants cause disease.

In summary, these data provide the first independent confirmation that biallelic *PRORP* missense variants can reduce mitochondrial tRNA processing in vitro and are associated with variable, overlapping pleiotropic phenotypes consistent with COXPD54. A possible association between phenotypic severity and mitochondrial tRNA processing deficit is starting to emerge, and further cases need to be defined to determine if robust genotype–phenotype correlations exist. Investigating alternate mechanisms of pathology may elucidate how variants with limited effect on mt-tRNA processing (p.Tyr365His) may cause disease.

### Supplementary information

Supplementary Material

## Data Availability

The *PRORP* variants were submitted to ClinVar (https://www.ncbi.nlm.nih.gov/clinvar/) (GenBank: NM_014672.4; accession numbers SCV002820061–SCV002820063).
