# Supplementary material for: Novel homozygous variants in PRORP expand the genotypic spectrum of combined oxidative phosphorylation deficiency 54
Source: Eur J Hum Genet. 2023 Aug 9;31(10):1190–4. doi: 10.1038/s41431-023-01437-2 (PMC10545766; doi:10.1038/s41431-023-01437-2)
Supplement: Supplementary file 1 — Supplementary Material [file 41431_2023_1437_MOESM1_ESM.pdf]

Supplemental Information

Materials and Methods

Variant identification methodology

For F1, exome sequencing (ES) was undertaken on a DNA sample from the proband by Macrogen Company (Macrogen Europe BV, Amsterdam, Netherlands). Whole-exome capture and enrichment were performed using SureSelect Human All Exon kit V6 (Agilent Technologies, Santa Clara, CA, USA). The enriched libraries were sequenced by Illumina Novaseq 6000 genome sequencing analyzer platform (Illumina, San Diego, CA, USA). ES data annotation, filtering, and interpreting were done as previously described (1).

For F2, DNA was enriched for the complete coding regions of most genes of the human genome using a proprietary capture system developed by GeneDx for next generation sequencing with CNV calling. The enriched targets were simultaneously sequenced with paired end reads on an Illumina platform. Bidirectional sequence reads were assembled and aligned to reference sequences based on NCBI RefSeq transcripts and human genome build GRCh37/UCSCS hg19. Using a custom-developed analysis tool (XomeAnalyzer) Ddata were filtered and analysed using XomeAnalyzer to identify sequence variants.

For F3, trio exome analysis was performed using an xGen Exome Research Panel v1.0 enrichment (IDT DNA), with paired end reads generated on a NOVASEQ 6000 (Illumina).

Respiratory chain activity assays and immunoblotting in fibroblasts

Respiratory chain complex activities were assessed in fibroblasts as described previously (2). Extraction of proteins and subsequent SDS-PAGE and immunoblotting was also performed on patient fibroblasts, as previously described (3), using primary antibodies against GAPDH (Proteintech, Rosemont, IL, USA:60004-1-Ig) and PRORP (Proteintech:20959-1-AP).

Mutagenesis of *PRORP*

The vector pET-28b(+) containing the *PRORP* cDNA sequence from amino acid 46 was mutagenised, as described previously (4). Oligonucleotides designed for mutagenesis are listed in Table S1, with altered bases highlighted. Following transformation of the mutagenesis reaction, individual colonies were cultured overnight, and plasmid DNA was extracted using a QIAprep Spin Miniprep Kit (Qiagen, Hilden, Germany). Successful mutagenesis was confirmed with Sanger sequencing using petup and T7 terminator primers (Eurofins Genomics, Ebersberg, Germany).

Expression and purification of recombinant TRMT10C, SDR5C1 and PRORP

pET-28b(+) plasmids containing PRORP wild-type and the individual variants listed in Table 1 were transformed into Rosetta 2 (DE3) E. coli cells (Novagen, Merck, Rahway, N.J., USA) and cultured in Overnight Express TB medium at 19°C (Novagen). TRMT10C and SDR5C1 were co-expressed in Rosetta 2 (DE3) cells (5). All proteins were purified by affinity chromatography utilising the 6x His-tag as previously described (5). Cells were lysed in a lysis/wash buffer comprised of 20mM Tris-Cl pH 7.4, 150mM NaCl, 0.1mM DTT, 0.02% Tween-20 (Sigma-Aldrich, St Louis, MO, USA), 20mM imidazole (Sigma) and 15% glycerol. His-tagged proteins were then eluted in lysis/wash buffer containing 250mM imidazole. Fractions were visualised via SDS-PAGE and ReadyBlue Protein Gel Stain solution (Sigma). Selected fractions were dialysed overnight at 4°C in dialysis buffer (20mM Tris-HCl pH 8, 200mM NaCl, 2mM DTT, 15% glycerol), centrifuged at 17,000 x g for 10 minutes at 4°C to remove insoluble proteins and frozen at -80°C.

Production of mitochondrial pre-tRNA transcripts

The pre-$tRNA^{Ile}$ from plasmid phI2 (6) was prepared by XbaI digestion of the plasmid, then *in vitro* transcription using the T7 RiboMAX Express Large Scale RNA Production System according to the

manufacturer's instructions (Promega, Madison, WI, USA). Following nucleic acid extraction with phenol:chloroform:isoamyl alcohol (Sigma), RNA was precipitated with ammonium acetate (Sigma) and ethanol, purified by washing with 95% ethanol and resuspended in water.

Mitochondrial pre-tRNA processing assay

Pre-tRNA processing assays were completed as previously defined (5,6). Once the reaction was initiated, samples were collected after 0 and 30 minutes and mixed 1:1 with a denaturing bromophenol blue formamide loading dye. Transcripts were resolved employing 6% acrylamide/8M urea gels. Gels were stained for 40 minutes with SYBR Green II RNA Gel Stain (Thermo Fisher Scientific, Waltham, MA, USA), diluted 1:10,000 and cleavage products were visualised with the LICOR Odyssey FC imaging system. Quantification of product intensity was accomplished with the Image Studio software, version 5.2.5. Assays were repeated independently at least four times, with data presented using GraphPad Prism 9 and error bars representing standard error about the mean. Statistical analysis was completed using GraphPad Prism 9, using one-way ANOVA to compare wild type to variants.

A

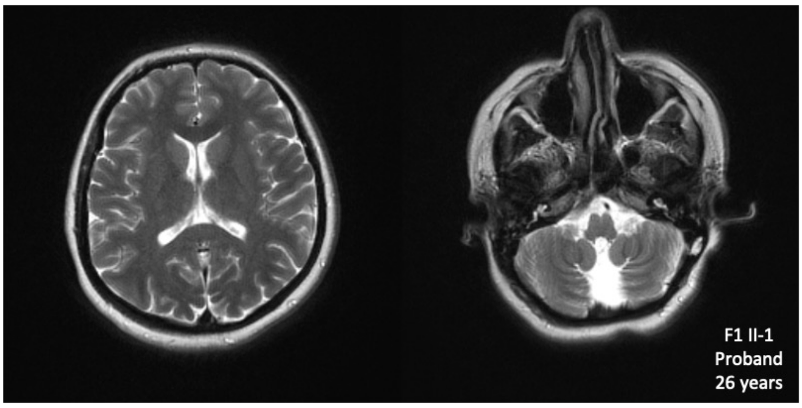

B

Test	Result	Follicular phase reference values
Estradiol (pg/mL)	33.21	18.9 – 246.7
Luteinising Hormone (mlu/mL)	8.94	2.4 – 12.6
Follicle Stimulating Hormone (mlu/mL)	6.73	3.5 – 12.5

C

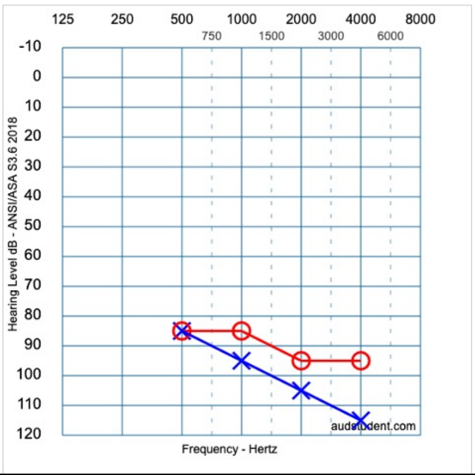

D

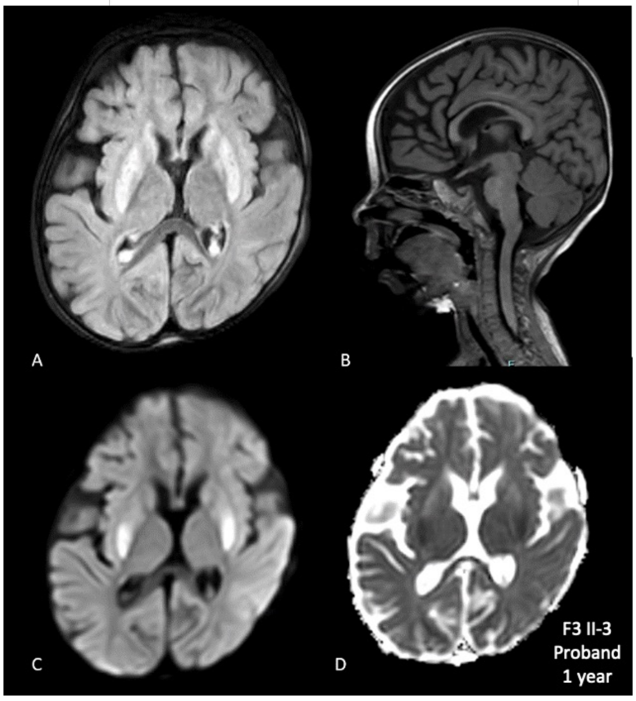

Figure S1: Additional clinical data for affected individuals in all families.
(A) Axial T2-weighted brain MR images taken of individual F1 II-1, exhibiting multifocal leukoencephalopathy and cerebellar atrophy (particularly in the vermis), respectively.
(B) Hormone profile for individual F1 II-1.
(C) Audiogram for individual F2 II-1, highlighting severe to profound bilateral sensorineural hearing loss in the proband with increasing severity at higher frequencies. Hearing level in the left ear is represented by blue crosses, and the right ear with red circles. Designed using the AudGen online tool (version 0.6.3) (https://audsim.com/audgenJS/audgenjs.html).
(D) MR images of individual F3 II-3.
A - Axial FLAIR showing bilateral symmetrical hyperintensities in the globus pallidus and putamen nuclei, and mildly in the head of the caudate nuclei.
B - Sagittal T1 highlighting a short corpus callosum with a thin body.
C (DWI) + D (ADC) - Shows bilateral symmetrical restricted diffusion in the globus pallidus, most prominent in the posterior parts. May be resultant of hypoxic ischaemic injury.
(FLAIR - fluid-attenuated inversion recovery, DWI - diffusion-weighted image, ADC - apparent diffusion coefficient).

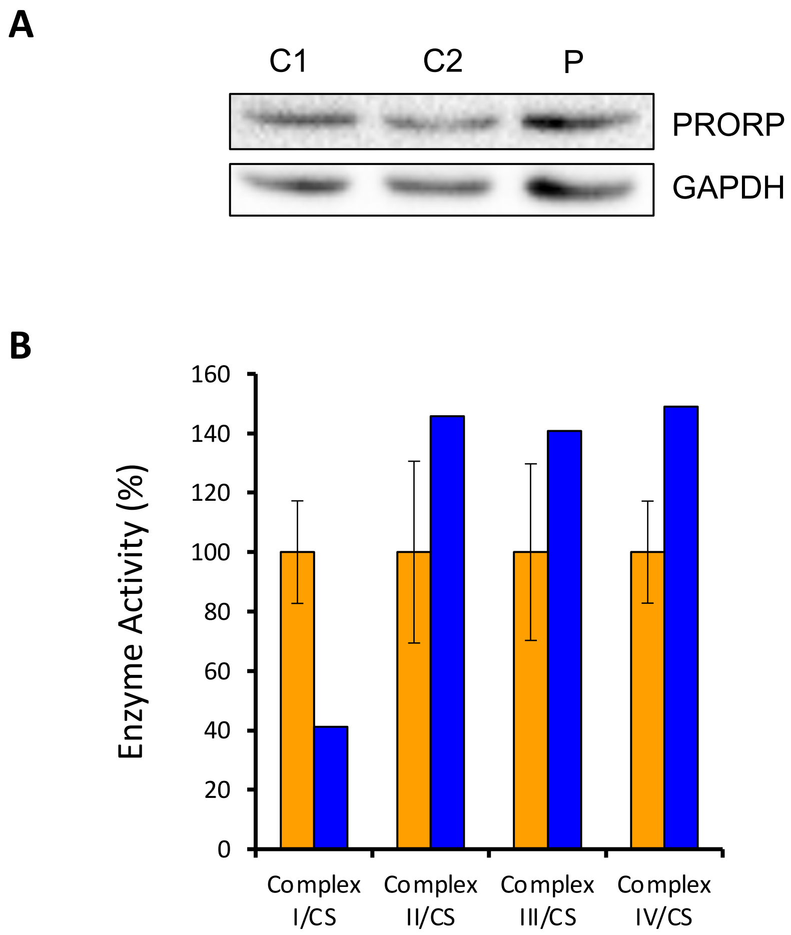

Figure S2: Analysis of PRORP expression and OXPHOS complex activities in F3 patient fibroblasts.
(A) Representative immunoblot comparing PRORP protein levels from F3 proband fibroblasts to two independent age-matched controls.

(B) Mitochondrial respiratory chain enzyme activities in control (orange) and *PRORP* patient (blue) fibroblast samples. Mean enzyme activities in control fibroblasts (n = 8) are set at 100%. Error bars represent the standard deviation.

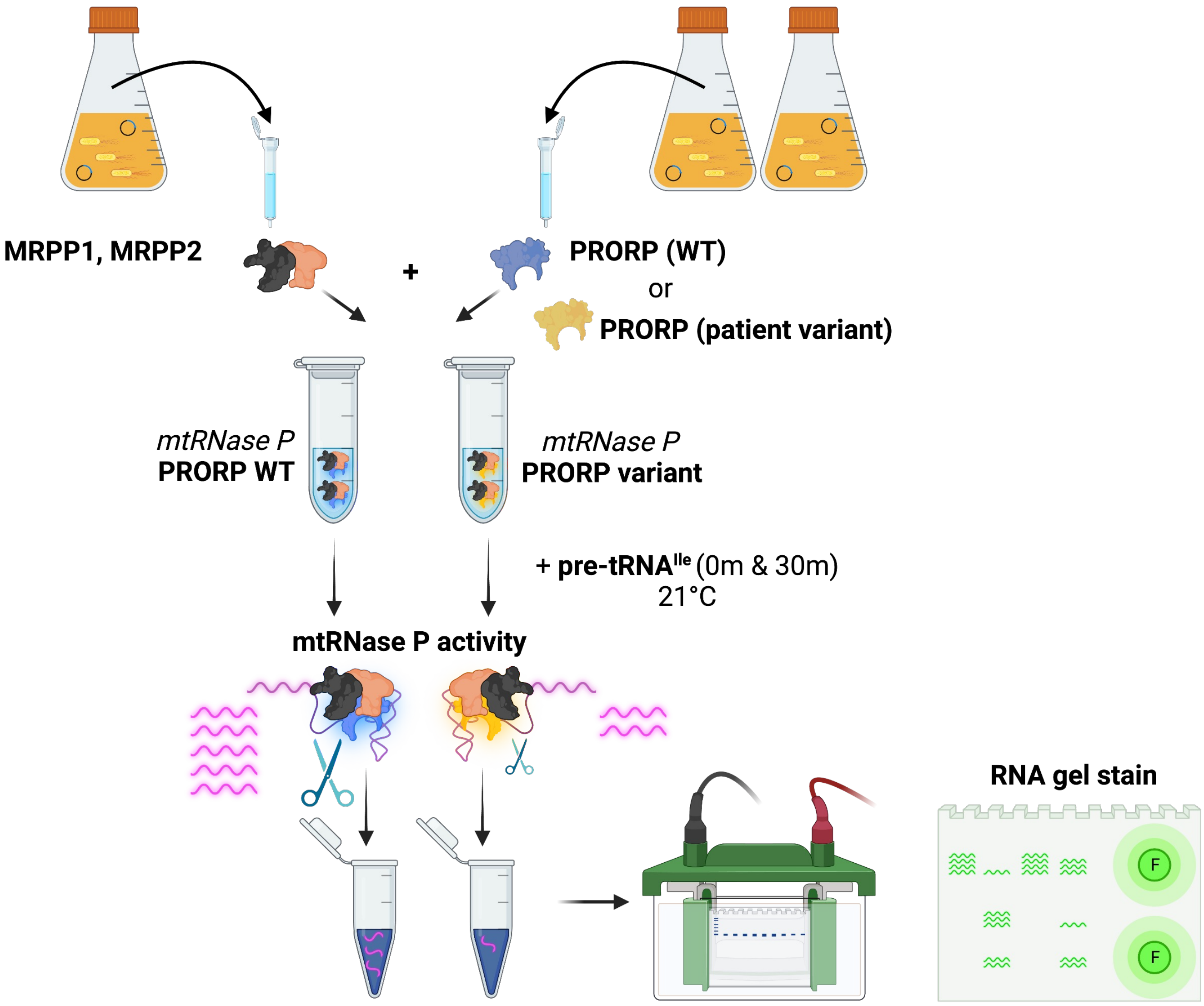

Figure S3: Schematic illustrating the mitochondrial tRNA processing assay. Recombinant MRPP1, MRPP2 and PRORP (WT or patient variant) were purified, then reconstituted to form the mtRNase P complex *in vitro*. *PRORP* variants can diminish mtRNase P activity, resulting in reduced 5'-end pre-tRNA processing, which is visualised on a denaturing urea gel to compare mtRNase P efficacy. Created with BioRender.com.

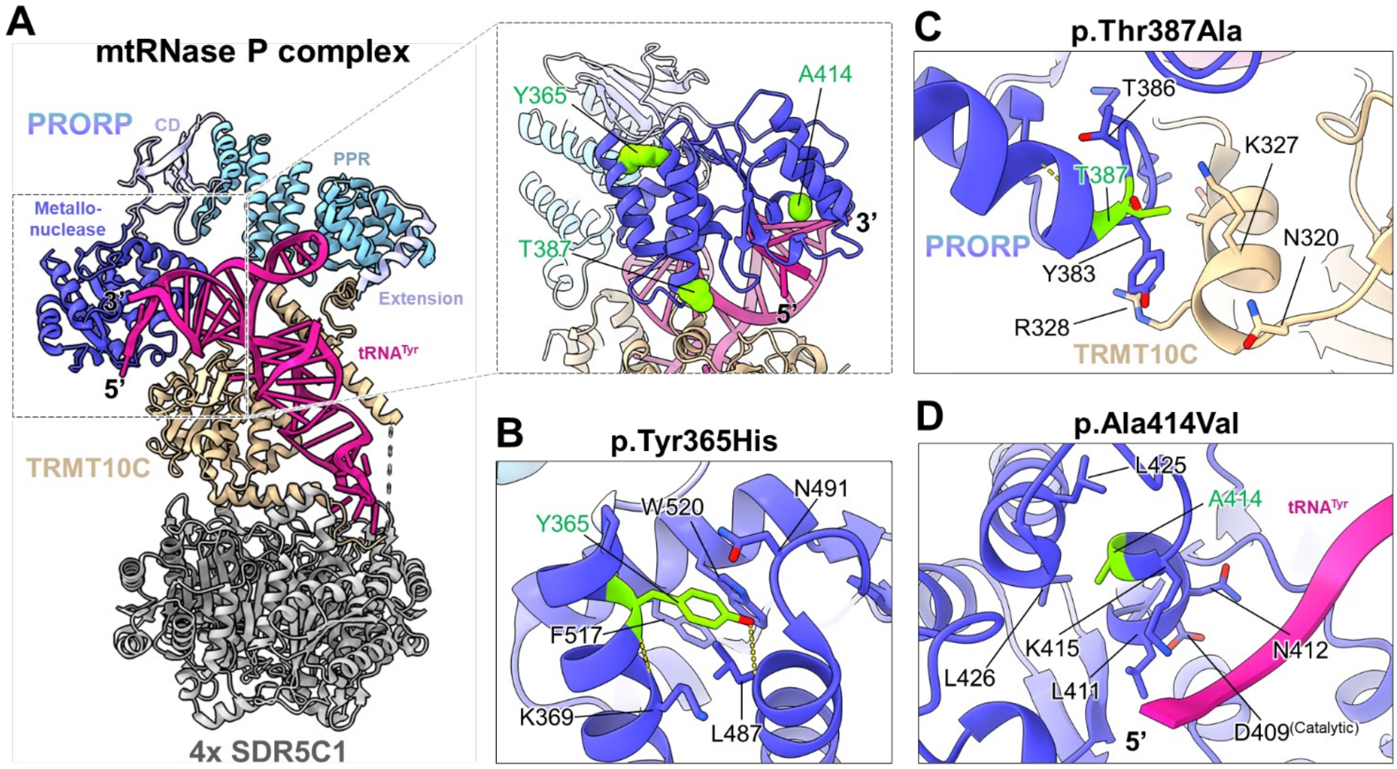

Figure S4: Structural analysis of *PRORP* variants based on the known mtRNase P complex.
(A) Cryo-electron microscopy structure of the mtRNase P complex (PDB:7ONU) consisting of a SDRC51 tetramer, a TRMT10C monomer, and a PRORP monomer engaged with a $tRNA^{Tyr}$ substrate. The individual domains of PRORP are labelled. Inset shows a zoom-in view of the metallonuclease domain of PRORP. The reported variants are labelled and highlighted in chartreuse.

(B) The location of the residue Tyr365. Substitution to histidine results in a similarly bulky side chain but could potentially remove a hydrogen bond. Mapping Tyr365 onto the known mtRNase P structure[7] shows that this residue is not located in the vicinity of the active site or any interaction regions.

(C) The location of the residue Thr387. This residue is located at the interaction site with TRMT10C. Its substitution to alanine may alter mtRNase P complex formation.

(D) The location of the residue Ala414. Substitution to valine will result in a bulkier sidechain and a possible clash with Leu426. This may result in altered interactions with the 5' end of the tRNA substrate. Recent structural studies have revealed that the adjacent residue Lys415 contacts the acceptor arm and leader nucleotides of the pre-tRNA substrate, helping to position the tRNA substrate into the active site[7]. This observation suggests that variants in this region of the metallonuclease domain could impair proper positioning and cleavage of pre-tRNAs.

Variant	Oligonucleotide sequence – 5′ to 3′
c.1093T>C (p.Tyr365His)	**GATTTTTCCCTTAAGACATTCATGTTCTTCTGGACTCAGC**
c.1159A>G (p.Thr387Ala)	**CTCTTAAGTTCCTGAGGCGCTGTCTTTCTGTACTGGTC**
c.1241C>T (p.Ala414Val)	**GAACTTTAGGAAACATTTTCACAACATTGAGACCATC**

Table S1: Oligonucleotide sequences used to modify *PRORP* ssDNA.

Variant	c.1093T>C p.(Tyr365His)	c.1159A>G p.(Thr387Ala)	c.1241C>T p.(Ala414Val)
Location	Chr14(GRCh38):g.35127537T>C	Chr14(GRCh38):g.35127603A>G	Chr14(GRCh38):g.35180743C>T
**SIFT**	Deleterious (0)	Deleterious (0)	Deleterious (0.01)
**Polyphen-2 (HumDiv)**	Probably damaging (0.994)	Possibly damaging (0.802)	Probably damaging (0.922)
**CADD**	Damaging (26.7)	Damaging (24.5)	Damaging (25)
**REVEL**	Tolerated (0.670)	Tolerated (0.508)	Tolerated (0.616)

Table S2: *In silico* analyses of the novel *PRORP* variants.

	Hochberg et al, F1	Hochberg et al, F2	Hochberg et al, F3	Hochberg et al, F4	This study, F1	This study, F2	This study, F3
PRORP variant(s)	c.1454C>T (**p.Ala485Val**)	c.1235A>G (**p.Asn412Ser**)<br>c.1301C>A (**p.Ala434Asp**)	c.1334G>A (**p.Arg445Gln**)<br>c.1197dupA (**p.Ser400IlefsX6**)	c.1261C>T (**p.Arg421Cys**)	c.1093T>C (**p.Tyr365His**)	c.1159A>G (**p.Thr387Ala**)	c.1241C>T (**p.Ala414Val**)
Affected family members	Three female siblings	One male	One male	Two siblings (one male & one female), their mother	One female	One male	One female
Hearing impairment	Bilateral, profound SNHL	Bilateral, mild-to-moderate SNHL (cookie-bite pattern)	Bilateral SNHL	No hearing impairments	No hearing impairment	Bilateral, severe-to-profound SNHL	NR (did not pass neonatal hearing screening test)
Fertility	Primary amenorrhea, HH, no ovarian tissue	NR	NR	No apparent abnormalities	No POI, but irregular menstruation and polycystic ovaries	NR	NR
Neurological findings	NR	No anomalies	White matter changes	White matter changes	White matter changes	No anomalies	White matter changes
Other characteristics	Mild intellectual disability	NR	Developmental delay, diffuse hypertonia, lactic acidosis	Seizures, learning disability	Mild intellectual impairment, gait abnormality	Developmental delay, spastic diplegia, truncal hypotonia	Developmental delay, lactic acidosis crisis, failure to thrive

Table S3: Comparison of clinical phenotypes observed with all reported *PRORP* variants to date.
NR = Not reported, HH = hypergonadotropic hypogonadism, POI = primary ovarian insufficiency.

References

1. Makrythanasis P, Maroofian R, Stray-Pedersen A, Musaev D, Zaki MS, Mahmoud IG, et al. Biallelic variants in KIF14 cause intellectual disability with microcephaly. Eur J Hum Genet. 2018;26:330–9.

2. Kirby DM, Thorburn DR, Turnbull DM, Taylor RW. Biochemical assays of respiratory chain complex activity. Methods Cell Biol. 2007;80:93-119.

3. Thompson K, Majd H, Dallabona C, Reinson K, King MS, Alston CL, et al. Recurrent De Novo Dominant Mutations in SLC25A4 Cause Severe Early-Onset Mitochondrial Disease and Loss of Mitochondrial DNA Copy Number. Am J Hum Genet. 2016;99:860-76.

4. Kunkel TA, Roberts JD, Zakour RA. Rapid and efficient site-specific mutagenesis without phenotypic selection. Methods Enzymol. 1987;154:367–82.

5. Hochberg I, Demain LAM, Richer J, Thompson K, Urquhart JE, Rea A, et al. Bi-allelic variants in the mitochondrial RNase P subunit PRORP cause mitochondrial tRNA processing defects and pleiotropic multisystem presentations. Am J Hum Genet. 2021;108:2195-2204.

6. Rossmanith W, Tullo A, Potuschak T, Karwan R, Sbis E. Human Mitochondrial tRNA Processing. J Biol Chem. 1995;270:12885–91.

7. Bhatta A, Dienemann C, Cramer P, Hillen HS. Structural basis of RNA processing by human mitochondrial RNase P. Nat Struct Mol Biol. 2021;28:713–23.
